# The speed of magnitude processing and executive functions in controlled and automatic number comparison in children: an electro-encephalography study

**DOI:** 10.1186/1744-9081-3-23

**Published:** 2007-04-30

**Authors:** Dénes Szũcs, Fruzsina Soltész, Éva Jármi, Valéria Csépe

**Affiliations:** 1 Centre for Neuroscience in Education, Faculty of Education; University of Cambridge, CB2 8PQ, UK; 2 Department of Psychophysiology; Research Institute for Psychology; Hungarian Academy of Sciences, Budapest, Hungary; 3 Department of Psychology, ELTE University, Budapest, Hungary

## Abstract

**Background:**

In the numerical Stroop paradigm (NSP) participants decide whether a digit is numerically or physically larger than another simultaneously presented digit. This paradigm is frequently used to assess the automatic number processing abilities of children. Currently it is unclear whether an equally refined evaluation of numerical magnitude occurs in both controlled (the numerical comparison task of the NSP) and automatic (the physical comparison task of the NSP) numerical comparison in both children and adults. One of our objectives was to respond this question by measuring the speed of controlled and automatic magnitude processing in children and adults in the NSP. Another objective was to determine how the immature executive functions of children affect their cognitive functions relative to adults in numerical comparison.

**Methods and results:**

The speed of numerical comparison was determined by monitoring the electro-encephalographic (EEG) numerical distance effect: The amplitude of EEG measures is modulated as a function of numerical distance between the to-be-compared digits. EEG numerical distance effects occurred between 140–320 ms after stimulus presentation in both controlled and automatic numerical comparison in all age groups. Executive functions were assessed by analyzing facilitation and interference effects on the latency of the P3b event-related potential component and the lateralized readiness potential (LRP). Interference effects were more related to response than to stimulus processing in children as compared with adults. The LRP revealed that the difficulty to inhibit irrelevant response tendencies was a major factor behind interference in the numerical task in children.

**Conclusion:**

The timing of the EEG distance effect suggests that a refined evaluation of numerical magnitude happened at a similar speed in each age group during both controlled and automatic magnitude processing. The larger response interference in children than in adults suggests that despite the similar behavioural profile of children and adults, partially different cognitive processes underlie their performance in the NSP. Further, behavioural effects in the NSP depend on interactions between comparison, facilitation/interference and response-related processes. Our data suggest that caution is needed when using the NSP to compare behavioural markers of the numerical processing skills of children and adults.

## Background

The human brain probably represents number meaning by an evolutionarily grounded magnitude representation [1-3]. Quantity discrimination studies suggest that the magnitude representation codes quantity similarly to the physical properties of the world, like luminosity, or length. The regularity guiding the discrimination of perceptual phenomena is described by Weber's law which states that discrimination performance depends on the ratio of the to-be-discriminated quantities. This law holds for numerosity discrimination as well: it is harder to discriminate quantities when they differ less (their ratio is closer to 1) relative to the case when they differ more (their ratio is further away from 1). This suggests that the magnitude representation interprets magnitude and numerosity in a continuous, non-discrete fashion. Weber's law is valid for both non-symbolic numerosities (e.g. dots) and symbolically presented numbers, and its consequence is the best established marker of the magnitude representation, the so-called "symbolic distance effect" (DE): Participants take more time and are less accurate when comparing Arabic digits representing closer quantities (e.g. 4 and 5) than distant quantities (e.g. 1 and 5) for both one [4] and two-digit [5] numbers. The interpretation of this effect is that numbers are transcoded into a ratio-sensitive analogue representation, i.e. the magnitude relations, or the meaning of symbolic numbers, is coded by the analogue system. Neuroimaging data suggest that the magnitude representation resides in the left and right horizontal intraparietal sulci (HIPS) of the human brain (for a review see [3]). Symbolic distance has been shown to correlate with parietal functional magnetic resonance imaging (fMRI) activation [6-10]. Further, as early as around 200 ms after stimulus onset, numerical DEs can be detected in the amplitude of event-related brain potentials (ERPs) over parietal electrode sites [6,11-15], as well as in the event-related spectral perturbation [14]. The electro-encephalography results suggest that numerical meaning is evaluated till/at around 200 ms after the presentation of digits.

The behavioural symbolic DE has been shown not only in adults but also in children from kindergarten to grade seven [12,16]. This suggests that magnitude relations are interpreted in a phenomenologically similar fashion in children and adults. However, children are slower in making these numerical comparisons than adults. This could be interpreted as showing that children access numerical information more slowly than adults. However, a seminal study by Temple and Posner [12] demonstrated that this explanation is probably incorrect. In one task subjects decided whether the visually presented digits 1, 4, 6 or 9 were smaller or larger than 5. Both children and adults showed a behavioural DE. The average reaction time (RT) was 480 ms for adults and 1495 ms for children. Contrary to the huge RT difference, both groups showed a DE in the amplitude of the ERPs over parietal electrode sites at around 200 ms after stimulus presentation. The authors suggested that 5 year-old children were able to access numerical information as fast as adults, but that the less developed response-organization abilities of children impeded their behavioural performance. This research suggests that the overtly controlled processing of numerical magnitude is already highly automatized in young children.

**Figure 1:**
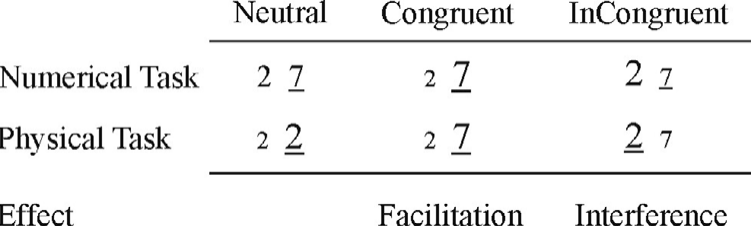
Examples of stimulus pairs used in the experiment. The correct response is underlined. Expected effects are also given in the bottom of the figure. Facilitation is the speed-up of the reaction time relative to the neutral condition. Interference is the slowing-down of the reaction time relative to the neutral condition.

In recent years, the degree of automatic access to task-irrelevant numerical information has been used as a measure of the development of numerical skills. Access to task-irrelevant numerical information has been measured using the so-called numerical Stroop paradigm (NSP) [17]. In this paradigm subjects compare simultaneously presented Arabic digits either on their physical or numerical magnitude (Fig. 1.). Numerical information is irrelevant in the physical comparison task (which is analogous to the colour naming task in the original Stroop paradigm [18]), yet adults consistently show interference effects, i.e. they slow down when the relative values of the relevant physical and the irrelevant numerical dimensions mismatch [[17,19,8,20]: see control group, [21,22]: see adult control groups,[23]]. This suggests that the irrelevant numerical information is automatically processed. Two behavioural cross-sectional studies investigated the NSP in children. Girelli et al. [21] found that the irrelevant numerical information influenced reaction times in grade 3, but not in grade 1. Rubinstein et al. [22] used a higher temporal sampling and found that irrelevant numerical information did not affect decision times at the beginning of grade 1, but already caused interference by the end of grade 1. Therefore, it seems that the automatization of extracting number meaning from symbols happens very early, during the first year of school.

Our study had three interrelated objectives. To date the Temple and Posner [12] study has been the only demonstration of an ERP DE in young children. First, we aimed to replicate the electroencephalographic (EEG) findings of Temple and Posner [12], and demonstrate that numerical magnitude is evaluated with a similar speed in both children and adults. This can be shown by detecting a significant DE in the amplitude of EEG measures with a similar timing in both children and adults. We expected that children will respond slower than adults, but the timing of the EEG DE will not differ in such extent in children and adults as the RT. Second, we aimed to extend earlier findings by examining whether the speed of magnitude processing is similar in children and adults both when numerical information is task-relevant (numerical comparison task of the Stroop paradigm) and when it is task-irrelevant (physical comparison task of the Stroop paradigm). Some studies found a facilitation/interference effect, but no DE, in the physical comparison task [19,20,22]. This was explained by arguing that only a crude (small/large) representation of number was retrieved in physical comparison but no refined evaluation of numerical meaning happened in this task [19]. From a developmental point of view it is of great interest whether an equally refined evaluation of magnitude happens in children and adults. If ERP DEs appear in children in both the numerical and physical comparison tasks, we can assume that a refined processing of numerical information happens in both tasks. Temple and Posner [12] have already demonstrated that behavioural measures may be less sensitive to cognitive processes than physiological measures, which are able to detect functional effects without the contamination of response-related processing. Therefore we expected to get a clear response to the above question by using ERPs.

Our third objective was to investigate the contribution of immature executive functions to numerical processing in the Stroop paradigm. In general, children's cognitive abilities are difficult to measure independently of attentional and behavioural immaturities relying on the long-lasting development of the prefrontal cortex. This may be especially so in the NSP which not only requires numerical skills but places severe demands on executive functions and response inhibition abilities, not yet fully developed in 6–12 year-old children [24]. This raises the possibility that facilitation and interference (see Fig. 1.) effects may rely on different stages of information processing in children and adults. It is important to have a clear view of the component processes of the NSP as it is widely used as a measure of the development of automatic number processing abilities. In fact, two recent fMRI studies of the NSP reported that 9–11-year-old children showed fMRI DEs predominantly in the frontal cortex [25,26]. These findings are in contrast with adult data where the strongest DEs are usually found in the parietal lobes [6-10]. These frontal DEs may indicate that frontal control processes play a more important role in numerical comparison in children than in adults in the NSP.

The excellent temporal resolution of ERPs permits the direct observation of the onset of facilitation of interference effects. More specifically, ERPs can determine whether facilitation and interference (for a review see [27]) appear at the level of stimulus or response processing in children and adults. Both Duncan-Johnson and Kopell [28] and Ilan and Polich [29] used the peak latency of the P3b ERP component to localize the temporal source of interference in the colour-word Stroop effect. Here we extended their method for the investigation of both facilitation and interference in the numerical Stroop paradigm. In simple tasks the P3b peak latency usually precedes RT and it is thought to reflect stimulus processing time [30] although cf. [31]. Independent from the exact nature of processes contributing to P3b latency, it is reasonable to assume that if facilitation and interference effects of similar magnitude appear both in the P3b latency and in the RT, the temporal locus of effects precede the peak latency of the P3b, or coincide with it. On the other hand, if larger facilitation and interference effects are present in the RT than in the P3b latency, we can assume that effects originate after the peak latency of the P3b, in a late phase of task-execution. Furthermore, response-related motor-cortex activity was explicitly examined by monitoring the Lateralized Readiness Potential (LRP [32]) which detects selective motor preparation before an overt response is given. In the above ways we investigated whether facilitation and interference is more related to stimulus or response processing in the NSP.

9 and 11-year-old children participated in our experiment. Previous data has clearly demonstrated that these children already show a facilitation/interference pattern in the NSP [21,22,25,26]. At the same time, developmental studies have demonstrated that children at these ages still have immature behaviour control abilities [24]. Further, fMRI studies of the NSP reported predominantly frontal DEs in children which is in contrast with adult data [25,26]. Therefore we hypothesized that the facilitation/interference pattern will be affected by the immature behaviour control abilities of the children. More specifically, we expected that while the pattern of behavioural effects will be similar in children and adults, response-related processing will play a more important role in children than in adults.

## Methods

### Participants

Three groups of subjects participated in the experiment. One group initially consisted of 16 Grade 3 children. 2 children were excluded from analysis (see results), this left 14 grade 3 children (mean age and standard deviation: 9.47 ± 0.37 years). Another group consisted of 16 Grade 5 children (11.55 ± 0.43 years). In the methods and results sections the grade 3 and 5 children will be referred to as "G3" and "G5" children. A third group consisted of 16 young adults (21.43 ± 2.44 years). All participants' behavioural data was analyzed. Ten subjects in each group were accepted for EEG data analysis after artefact filtering. No EEG analysis was done before artefact rejection. Participants' mean age and standard deviations: G3: 9.53 ± 0.36; G5: 11.46 ± 0.52; Adults: 21.6 ± 2.95. All children were recruited from the same two classes of a local public school in Budapest (Hungary). Children belonged to working class or middle class families. Adults were recruited by advertisement. No participant was reported to have any cognitive or neurological problems. All subjects or their parents gave written informed consent. Children were rewarded by sweets and book tokens, adults received payment for participation. The study was approved by the institutional ethics committee.

All children participants were reported to be normally developing. This was confirmed by determining their IQ and general calculation abilities. The IQ was determined by the Snijders-Oomen Nonverbal Intelligence Scale for Young Children. None of the children were reported to have age-inappropriate calculation problems by their parents and teachers. Nevertheless, the general arithmetic abilities of children were tested by the following tasks: 12 dot counting problems (5–10 dots), 12 one digit additions, 8 problems testing the knowledge of arithmetic rules, 5 story problems (1–1 addition, subtraction, division, multiplication and one problem with multiple operations), 12 subtractions. Harder problems were selected for the grade 5 than for the grade 3 children according to age-appropriateness.

### Experimental stimuli and task

Stimuli were pairs of Arabic digits shown in the middle of a 17-inch computer screen. There were 12 possible pairs of numbers (2–7, 3–8, 7–2, 8–3, 2–3, 7–8, 3–2, 8–7, as well as 2–2, 3–3, 7–7 and 8–8). There were two experimental tasks (Task factor). In the numerical comparison task subjects were instructed to decide which item of the pair was numerically larger than the other one. In the physical comparison task subjects decided which item of the pair was physically larger than the other one. Subjects signalled their decision by pressing a response button on the side (left or right) where they detected the numerically or physically larger digit (Response Hand factor). The numerical and physical dimensions of digits could be neutral, congruent or incongruent with each other (Congruency factor; see Fig. 1). The numerical distance (Distance factor) between the items of the pairs was 1 or 5 in all conditions, except in the neutral condition of the physical comparison task, where it was zero. Possible stimulus pairs were used in equal proportions. Possible stimulus pairs were randomized within blocks so that they could not be repeated in three consecutive trials.

Each trial began with the drawing of an eye shown for 500 ms. Subjects were instructed to blink if needed when they saw this fixation sign. After 500 ms, a pair of stimuli were shown for maximum 3 seconds, or until the subject responded. The stimuli were followed by a pause of 500 ms. Physically small stimuli had a font-size of 40, physically large stimuli had a font-size of 50, neutral stimuli had a medium font-size of 45. Only one level of physical distance was used, in order to be able to collect the number of epochs guaranteeing an adequate signal to noise ratio for EEG analysis. The viewing distance was approximately 80 cm. Stimulus pairs subtended a horizontal view angle of 2.26°. The vertical view angle was 1.13° in stimulus pairs with a small and large size stimulus, and 0.94° when two medium sized stimuli were presented. There were 5 blocks of 48 (altogether 240) stimuli per each task. Tasks were preceded by 24 practice stimuli. Half of the subjects participated first in the numerical task and than in the physical task. The order of task presentation was the opposite for the other half of the subjects.

### Behavioural analysis

After an initial assessment of all experimental trials receiving a correct response within 2500 ms, only trials correctly responded within 300–1600 ms were analyzed. In an initial analysis reaction times measured in individual trials were pooled across all subjects for each subject group, and the distributions of RTs in the first and the second half of the experiment were compared for all congruency conditions. Experimental time was taken into consideration in the factor structure of one analysis as a Time factor: trials acquired in the first vs. in the second half of the experiment. Effects in the two tasks were first compared by a Task × Group (numerical vs. physical) × Hand (left vs. right) × Time × Congruency (Neutral, Congruent and Incongruent) × Distance (1 vs. 5) ANOVA. As the numerical distance between digits was always zero in the neutral condition of the physical task, half of the trials in this condition were assigned to the distance 1 condition, and the other half to the distance 5 condition [22]. Second, focusing on the developmental pattern of effects, Group × Hand × Time × Congruency × Distance ANOVAs were run separately for both tasks. Further, In order to be able to better compare our results to earlier work [21,22] groups were tested separately by Hand × Time × Congruency × Distance ANOVAs separately for both tasks. In the ANOVAs run separately for the two tasks, the DE in the physical task was tested using data from the congruent and incongruent conditions only (as numerical distance was always zero in the neutral condition). The DE in the numerical task was tested using data from all three levels of congruency. Other effects were tested using the whole factor structure. In subjects accepted for EEG analysis the focused assessment of congruency effects was done by Task × Congruency ANOVAs. This analysis was run because Congruency effects were compared in the RT and in the P3b peak latency (see later). Therefore the factor structure was simplified in order to provide a good signal to noise ratio for the cells in the EEG analysis. Facilitation and interference effects were studied by examining congruent vs. neutral (Facilitation) and incongruent vs. neutral (Interference) Tukey post-hoc comparisons from single factor Congruency ANOVAs in each task. Further, Facilitation (Congruent-Neutral) and Interference (Incongruent-Neutral) values were also directly computed and two-tailed t-tests were run on these values to investigate whether they significantly deviated from zero. In order to investigate whether the pattern of facilitation and interference changed across groups, facilitation and interference effects in the RT and in P3b were studied by Group × Measure (RT vs. P3b peak latency) ANOVAs separately for facilitation and interference in both tasks. These will be described under the section on ERP analysis.

Greenhouse-Geisser correction was done in all behavioural and physiological ANOVAs as necessary. Original F and df, epsilon (ε), and corrected p values are reported.

### EEG acquisition

A BrainAmp amplifier, the BrainVision Recorder program and EasyCap electrode-caps were used for data acquisition, with the following standard electrode sites according to the international 10–20 system: Fp1, Fp2, F9, F7, F3, Fz, F4, F8, F10, Fc5, Fc1, Fc2, Fc6, T9, T7, C3, Cz, C4, T8, T10, Cp5, Cp1, Cp2, Cp6, P9, P7, P3, P4, P8, P10, O1 and O2. Voltage was referenced to Pz. The data was sampled at 500 Hz, using an online bandpass filter of 70 Hz, and later offline-filtered for 0.3–47 Hz. The data was recomputed to average reference and baseline-corrected relative to the -100 to 0 ms interval before stimulus onset. The reference electrode was re-used as electrode Pz. Therefore data on 33 electrodes was available. Epochs containing ocular artefacts (monitored visually at Fp1, Fp2, F9 and F10), and epochs containing voltage deviations larger than ±80 μV relative to baseline at any of the electrodes were rejected. Data analysis was done in Matlab, EEGLab [33], and Statistica 6.0.

### ERP analysis

The DE was examined using trials from the congruent and incongruent conditions. First, we used point-by-point Distance × Task ANOVAs (p < 0.025) separately in each group to identify significant DEs in the 100–400 ms interval at parietal electrode sites (P7, P3, P4, and P8). Intervals containing at least 8 consecutively significant points were considered significant. The mean amplitude of intervals found significant by point-by-point testing at parietal electrodes was subjected to Distance × Task ANOVAs. In a following analysis, the topography of the DE was assessed by running point-by-point Distance × Task ANOVAs on the amplitude at all 33 electrodes (p < 0.025; threshold: 8 consecutively significant points). The overall topography of the DE was approximated by computing distance 1 minus distance 5 difference potentials, and measuring the mean amplitude of the 140–180, 180–240, and 240–320 ms intervals over electrodes f7, f8, f3, f4, t7, t8, c3, c4, p3, p4, p7 and p8. Topographic comparisons were done by entering mean amplitude values of the above intervals into Group × Electrode ANOVAs separately for each task. When the Group × Electrode interaction was significant, Group × Location (frontal, central, parietal) × Hemisphere (left vs. right) × Extremity (lateral vs. midline electrodes) ANOVAs were run. Statistics were done on microvolt values.

The peak latency of the P3b ERP component was determined by measuring the latency of the maximum amplitude sampling point between 200–1000 ms in single trials at electrode Pz. The average latency of the P3b for each subject was computed form the single trial data. The peak latency of the P3b was analyzed by Task × Congruency ANOVAs run separately for each group. Facilitation and interference effects were studied by examining congruent vs. neutral (Facilitation) and incongruent vs. neutral (Interference) Tukey post-hoc comparisons from single factor Congruency ANOVAs in each task. Further, Facilitation (Congruent-Neutral) and Interference (Incongruent-Neutral) values were also directly computed and two-tailed t-tests were run on these values to investigate whether they significantly deviated from zero.

In order to investigate whether the pattern of facilitation and interference changed across groups, facilitation and interference effects in the RT and in P3b were studied by Group × Measure (RT vs. P3b peak latency) ANOVAs separately for facilitation and interference in both tasks. The difference between P3b latency and RT in each group was tested by examining relevant Tukey contrasts from Group × Measure (P3b vs. RT) × ANOVAs. The mean amplitude of the P3b in grand-average ERPs was examined in each 100 ms long interval between 500–1000 ms by Task × Congruency ANOVAs, separately for each group.

The LRP was computed as proposed by Coles [32]:

[(C4 - C3) _LEFT HAND response _+ (C3 - C4) _RIGHT HAND response _]/2,

where C3 and C4 denote the amplitude of the ERPs at electrodes C3 and C4. According to this convention, in adults a negative LRP indicates a correct response tendency, whereas a positive LRP indicates an incorrect response tendency. The deviation of LRPs from zero was tested by point-by-point two-sided one-sample t-tests against zero (p < 0.05) run in each condition [34]. Between-condition differences were tested by point-by-point 2-sample t-tests (p < 0.05) run between Congruent-Incongruent, Neutral-Incongruent and Neutral-Congruent conditions separately for both tasks. Intervals with at least 8 consecutively significant points were considered significant.

### Event-related spectral perturbation (ERSP) analysis

Unlike the ERP, which contains only information phase-locked to stimulus presentation, the ERSP contains information both phase-locked and not phase-locked (both evoked and induced activity) to stimulus presentation [35]. Therefore it is a more complete representation of the event-related EEG activity than the ERP. Further, the ERSP is able to differentiate between effects happening at different frequency components of the EEG. Here, time-frequency decomposition was performed by short-time Fourier transform in EEGLab [33]. Epochs from the incongruent and congruent conditions were used for investigating the ERSP DE. Artifact rejection parameters were the same as for ERP analysis, and the -100 ms to 0 ms interval served as the baseline for the artifact rejection only. Sampling points of baseline-uncorrected epochs between -100 and 600 ms relative to stimulus onset were used for ERSP analysis. A sliding temporal window of 128 points was applied 200 times providing output frequency bins at about 2 Hz steps, and output times between -36 to 536 ms with a resolution of 2.87 ms. DEs were detected by within-subject Distance × Task ANOVAs run separately for each group at each point of the time-frequency landscape. To compensate for intensive multiple testing a conservative (p < 0.005) significance level was used. The analysis focused on 50 sampling points (150–300 ms) × 23 frequency bins (2–45 Hz). Therefore at a p level of 0.005, 5.75 (50 × 23 × 0.005) points could be detected as significant due to chance at each electrode. This is considerably less than the number of points interpreted as showing significant effects (see later).

## Results

Accuracy was at ceiling level in all groups in all conditions in the experimental task (overall accuracy: G3: 99.63 ± 1.92; G5: 99.80 ± 1.49; Adults: 99.76 ± 1.52). Two G3 children were excluded from the sample because of too slow RT (mean + 3 standard deviations). This left 14 subjects in G3, and 16 subjects in G5 and in Adults for the behavioural analysis. In order to remove outliers only trials receiving a correct response between 300–1600 ms were kept for analysis. This removed less than 0.4% of the data in each group (G3: 24 trials; G5: 9 trials; Adults: 1 trial). The initial assessment of pooled reaction times showed that the distributions of RT were very similar in both the first and second part of the experiment in all groups. Further, the relative positions of the distribution of reaction times in the neutral, congruent, and incongruent conditions did not change substantially from the first to the second part of the experiment.

### IQ and calculation abilities

Considering all G3 and G5 children the IQ was 110 ± 13 in G3 and 111 ± 14 in G5 (group difference: n.s. /p > 0.9/). Considering only children accepted for EEG analysis the IQ was 110 ± 10 in G3 and 112 ± 14 in G5 (group difference: n.s. /p > 0.8/). In the calculation tests children committed 0–3 sporadic errors in different problems types. There were 49 problems. The range of the percent of correct solutions in G3 was 88–100% (0–6 errors), and in G5 this range was 82–100% (0–9 errors). This confirms that none of the child participants had arithmetic difficulties.

### Experimental task: behavioural data

**Figure 2:**
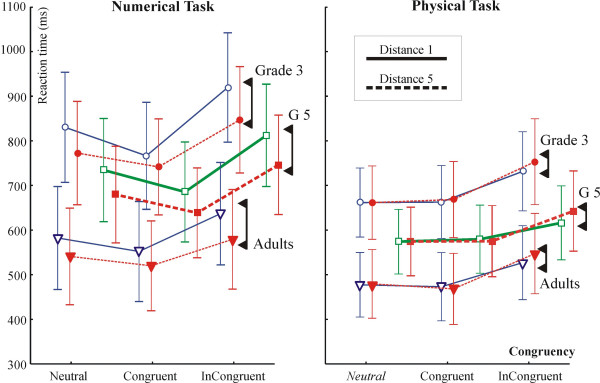
Reaction times in the numerical and physical comparison tasks for all 46 subjects. Note that the numerical distance was zero in the neutral condition of the physical comparison task (italics).

All purely behavioural analyses were done for all 46 subjects, and than separately for the subset of 30 subjects accepted for EEG analysis after artefact filtering. In terms of statistical effects the two series of behavioural analyses yielded identical results. When both EEG subjects' and all subjects' data was analyzed, results for subjects included in the EEG analysis are described first, and than results for all subjects are given in curly brackets ("{}"). The RTs for EEG subjects are given in Table 1, RTs for all 46 subjects are depicted in Figure 2.

**Table 1:** Reaction time for subjects included in the EEG analysis. (A) Reaction time in the numerical comparison task. (B) Reaction time in the physical size comparison task.

	**Neutral**		**Congruent**		**Incongruent**	
	**D1**	**D5**	**D1**	**D5**	**D1**	**D5**
**A.**						
**G3**	822 ± 61	761 ± 57	752 ± 59	727 ± 53	907 ± 61	834 ± 59
**G5**	727 ± 57	675 ± 54	697 ± 56	654 ± 50	810 ± 57	749 ± 55
**GA**	579 ± 57	531 ± 54	551 ± 56	509 ± 50	629 ± 57	572 ± 55
**B.**						
**G3**	631 ± 38	631 ± 41	635 ± 40	638 ± 42	696 ± 44	712 ± 48
**G5**	583 ± 36	583 ± 38	581 ± 38	582 ± 40	624 ± 41	647 ± 45
**GA**	468 ± 36	467 ± 38	459 ± 38	454 ± 40	511 ± 41	538 ± 45

#### Overall effects

According to a Task × Group × Hand × Time × Congruency × Distance ANOVA older participants responded faster than younger ones (Group: F(2,27) = 16.66; p < 0.0001 {F(2,43) = 27.81; p < 0.0001}). The physical task was responded 113 {106} ms faster than the numerical task (F(1,27) = 46.12; p < 0.0001 {F(1,43) = 66.89; p < 0.0001}). The overall RT was 15 {10} ms faster during the second half of the experiment than during the first half (F(1,27) = 10.33; p = 0.0033 {F(1,43) = 5.77; p = 0.0206}). There was an overall DE (F(1,27) = 66.74; p < 0.0001 {F(1,43) = 81.9; p < 0.0001}), a Distance × Task interaction (F(1,27) = 122.9; p < 0.0001 {F(1,43) = 142.3; p < 0.0001}), a Congruency effect (F(2,54) = 211.6; ε = 0.702; p < 0.0001; {F(2,86) = 331.3; ε = 0.839; p < 0.0001}), and a Congruency × Task interaction (F(2,54) = 15.8; ε = 0.812; p < 0.0001 {F(2,86) = 23.3; ε = 0.966; p < 0.0001}). The sources of these effects are explained in detail below.

#### Distance effect

According to a Group × Hand × Time × Congruency (neutral, congruent and incongruent) × Distance ANOVA there was a DE (F(1,27) = 119.78; p < 0.0001 {F(1,43) = 159.72; p < 0.0001}) in the numerical task: The RT was 51 {50} ms faster in condition Distance 5 than in Distance 1. The Distance × Group interaction was insignificant (F<1; p = 0.94 {p = 0.42}). In the physical task the DE was tested using only the congruent and incongruent conditions (numerical distance was zero in the neutral condition). There was a reversed DE in the physical task: the RT was 11 {11} ms slower in condition Distance 5 than in Distance 1 (F(1,27) = 6.26; p = 0.0186 {F(1,43) = 8.30; p = 0.0061}). The Distance × Group interaction was insignificant (F<1; p = 0.97 {p = 0.81}).

In order to be able to better compare our results to earlier work [21,22] groups were tested separately by Hand × Time × Congruency × Distance ANOVAs separately for both tasks. DEs and effect sizes in milliseconds are given in Table 2. The DE was significant in all groups in the numerical task. In the physical task the DE was significant only in adults but not in children. There was a Distance × Congruency interaction in adults (F(2,18) = 10.45; ε = 0.625; p = 0.0056 {F(2,30) = 5.38; ε = 0.690; p = 0.0217}). The interaction appeared because the reversed DE was much more expressed in the incongruent than in the congruent condition. It is noteworthy that contrary to the lack of a significant reversed DE in children, the effect size in milliseconds was very similar in children and adults. The differential pattern of the DE in the two tasks is very well seen in Fig. 2.

**Table 2:** Distance and Congruency effects. For the distance effect the effect size ("effect") is given as distance 1 minus distance 5 values in milliseconds. For the physical task both the overall effect size and the effect size for the incongruent condition is given, separated by "/". (A) Effects for all 46 subjects. (B) Effects for the 30 subjects whose EEG data was analyzed.

**A. All Subjects**		**Numerical Task**			**Physical Task**		
		**Grade 3**	**Grade 5**	**Adults**	**Grade 3**	**Grade 5**	**Adults**
Distance effect	df	(1,13)	(1,15)	(1,15)	(1,13)	(1,15)	(1,15)
	F	85.2	38.98	71.92	--	--	5.38
	p	<0.0001	0.0003	<0.0001	n.s.	n.s.	0.0348
	effect	52	56	44	-13/-22	-8/-26	-7/-20
Congruency effect	df	(2,26)	(2,30)	(2,30)	(2,26)	(2,30)	(2,30)
	F	135.81	62.94	78.28	32.41	37.07	56.23
	ε	0.881	0.774	0.943	0.709	0.741	0.652
	p	<0.0001	<0.0001	<0.0001	<0.0001	<0.0001	<0.0001

**B. EEG Subjects**		**Numerical Task**			**Physical Task**		
		**Grade 3**	**Grade 5**	**Adults**	**Grade 3**	**Grade 5**	**Adults**

Distance effect	df	(1,9)	(1,9)	(1,9)	(1,9)	(1,9)	(1,9)
	F	48.45	25.84	67.57	--	--	5.49
	p	<0.0001	0.0006	<0.0001	n.s.	n.s.	0.0437
	effect	53	52	49	-7/-18	-10/-23	-11/-26
Congruency effect	df	(2,18)	(2,18)	(2,18)	(2,18)	(2,18)	(2,18)
	F	94.3	89.63	58.07	27.27	29.66	39.11
	ε	0.642	0.913	0.981	0.581	0.798	0.596
	p	<0.0001	<0.0001	<0.0001	0.0002	<0.0001	<0.0001

#### Congruency effect: facilitation and interference

**Figure 3:**
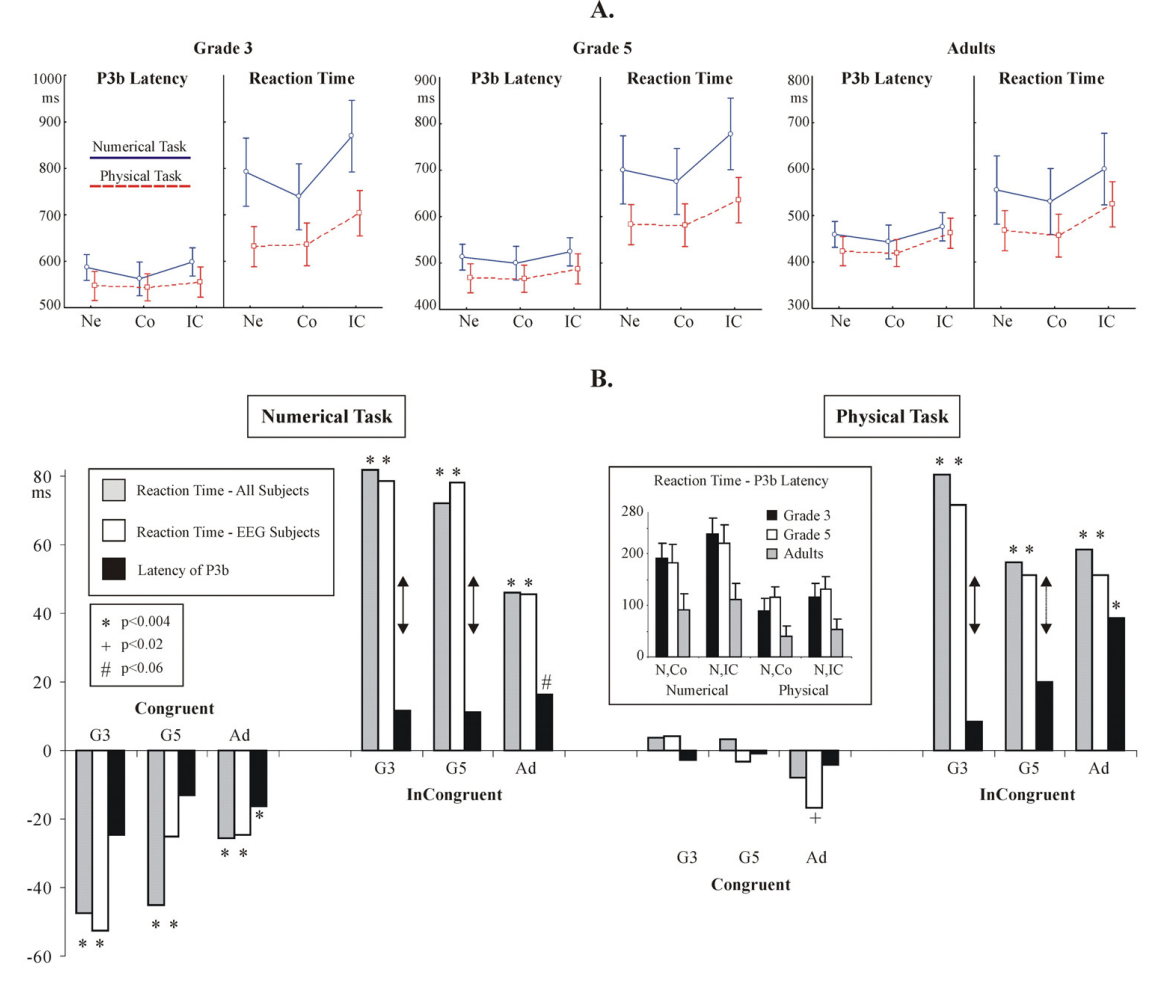
(A) Congruency effects in the P3b latency and in reaction time in subjects accepted for EEG analysis. Conditions are represented on the X axis: Ne: neutral. Co: Congruent. IC: Incongruent. (B) Facilitation and interference effects on the reaction time (RT) and on the latency of the P3b ERP component in grade 3 (G3), grade 5 (G5), and in adults (Ad). The RT of all 46 subjects and of the 30 of subjects accepted for EEG analysis is shown separately. Facilitation and interference values were computed as Congruent minus Neutral, and Incongruent minus Neutral values. The deviation of the difference values from zero was tested by two-tailed one-sample t-tests against zero. Results of these tests are represented by "*", "+" and "#" signs (see legend). Double-headed arrows denote the cases when interference effects on the latency of the P3b and the on RT were significantly different (see text for details). The insert shows the difference between the RT and P3b latency in milliseconds (with standard errors). X axis of the insert: "N,Co": the average of the neutral and congruent conditions. "N,IC": the average of the neutral and incongruent conditions.

Congruency effects are shown in Figure 3A. According to a Group × Hand × Time × Congruency × Distance ANOVA, in the numerical task there was a congruency effect (F(2,54) = 239.39; ε = 0.863; p < 0.0001 {F(2,86) = 245.30; ε = 0.954; p < 0.0001}), and a Group × Congruency interaction (F(4,54) = 7.65; ε = 0.863; p < 0.0002 {F(4,86) = 6.64; ε = 0.954; p < 0.0002}). In the physical task there was a Congruency effect (F(2,27) = 100.95; p < 0.0001. {F(2,86) = 118.11; ε = 0.711; p < 0.0001}), and no Group × Congruency interaction. For comparison with earlier results Hand × Time × Congruency × Distance ANOVAs were run for all groups separately for both tasks. The main effect of congruency was significant in all groups in both tasks (see Table 2.).

In order to enable direct comparisons with Congruency effects in the P3b latency (see later) Congruency effects were further examined by Task × Congruency (Neutral, Congruent, Incongruent) ANOVAs run in EEG subjects. Congruency was a significant factor in each group (G3: F(2,18) = 80.32; ε = 0.600; p < 0.0001. G5: F(2,18) = 101.2; ε = 0.821; p < 0.0001. Adults: F(2,18) = 74.96; ε = 0.732; p < 0.0001.). There were Task × Congruency interactions in children but not in adults (G3: F(2,18) = 14.85; ε = 0.576; p = 0.0023. G5: F(2,18) = 10.27; ε = 0.873; p = 0.0018.).

#### Facilitation and interference: the effect of age

Facilitation and interference effects were tested by Tukey contrasts from single factor Congruency ANOVAs in each task and group. Statistical results are given in Table 3A., and facilitation and interference effects are visualized in Figure 3B. In the numerical task both facilitation and interference were significant in all groups. In the physical task, only interference was significant in all groups. Group × Congruency ANOVAs run on facilitation and interference values revealed that both facilitation (F(2,27) = 6.39; p = 0.0051. Post-hoc Tukey p: G3 vs. Adults: p = 0.0122. G3 vs. G5: p = 0.0122) and interference (F(2,27) = 4.67; p = 0.0180. G3 vs. Adults: p = 0.0345. Adults vs. G5: p = 0.0346) differed by group in the numerical task. In the physical task, there was a marginal group effect in facilitation (F(2,27) = 2.84; p = 0.075. G3 vs. Adults: p = 0.0636.), and no group effect in interference. These interactions are in-line with interactions mentioned in the preceding paragraph.

**Table 3:** 

		**A. Reaction Time**			**B. P3b peak latency**		
		
		**Grade 3**	**Grade 5**	**Adults**	**Grade 3**	**Grade 5**	**Adults**
**Numerical Task**	F	91.50	90.01	57.31	n.s.	n.s.	14.74
F(2,18)	ε	0.626	0.921	0.985	---	---	0.711
Congruency	p	<0.0001	<0.0001	<0.0001	---	---	0.0010
Facilitation	p	0.0002	0.0152	0.0040	---	---	0.0368
Interference	p	0.0001	0.0001	0.0002	---	---	0.0355
**Physical Task**	F	26.92	30.00	38.44	n.s.	n.s.	16.29
F(2,18)	ε	0.586	0.800	0.592	---	---	0.626
Congruency	p	0.0002	<0.0001	<0.0001	---	---	0.0012
Facilitation	p	0.89	0.98	0.42	---	---	0.88
Interference	p	0.0002	0.0002	0.0002	---	---	0.0006

### Experimental task: EEG data

#### Distance effect: ERPs at parietal electrodes

**Figure 4:**
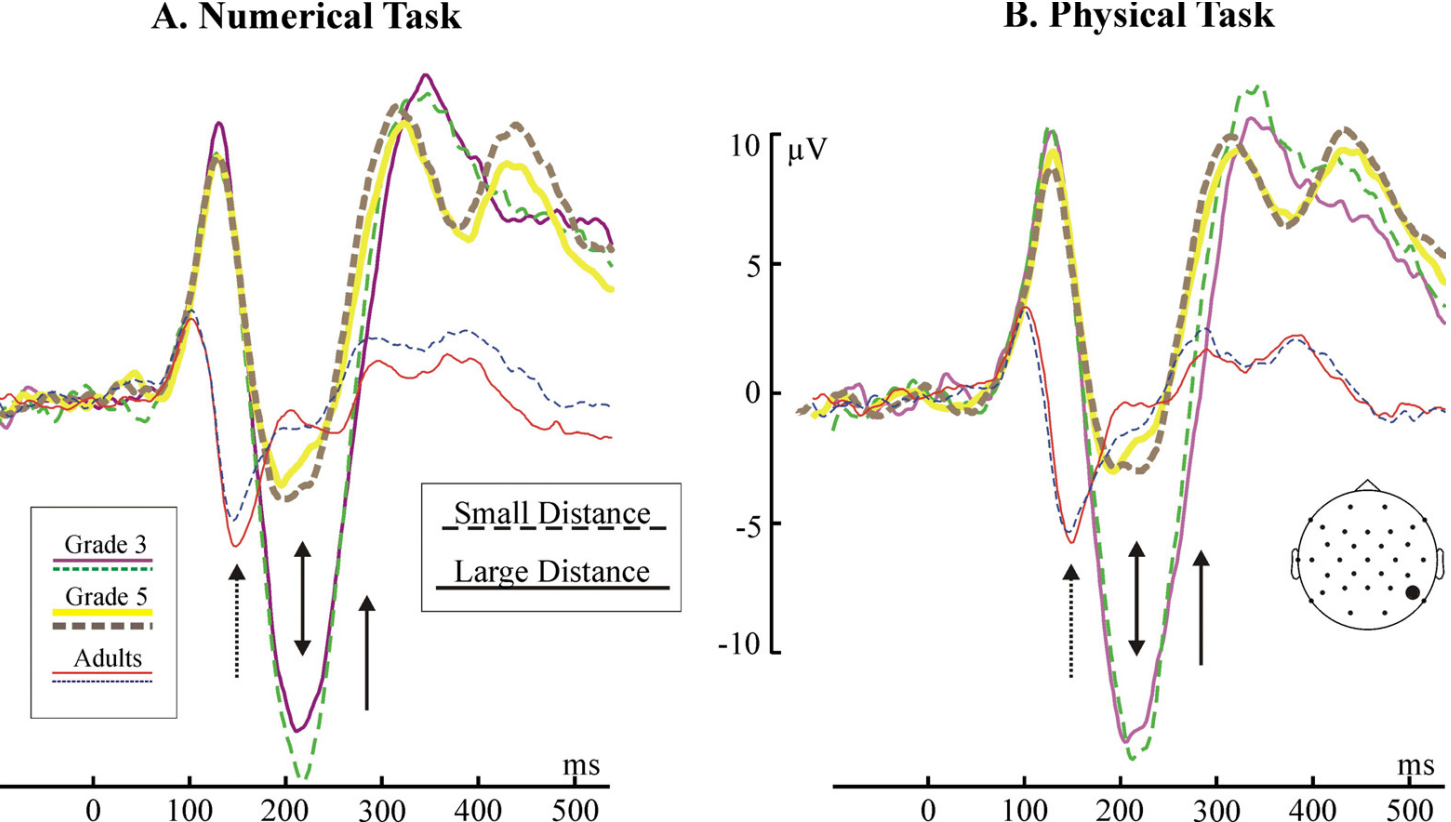
The distance effect in event-related potentials (ERPs) at the parietal electrode P8. Vertical arrows denote distance effects at around 200 ms. The insert denotes the position of electrode P8. (Small distance: distance 1; large distance: distance 5).

Temple and Posner [12] focused on DEs over parietal electrode sites. Therefore, first we analyzed ERP DEs over parietal electrodes (P7, P3, P4 and P8). DEs were consistently detected in both conditions at the right parietal electrode P8. DEs at electrode P8 are shown in Fig. 4. Statistical results for the time intervals with significant ERP DEs at electrode P8 are given in Table 4. There were no Distance × Task interactions at electrode P8 in children. There were Distance × Task interactions in adults. Between 145–165 ms the DE was stronger in the Numerical (Tukey p = 0.0002) than in the Physical task (p = 0.0241). Between 190–210 ms the DE was marginally significant in the numerical task (p = 0.0636), and it was stronger in the physical task (p = 0.0010). The earliest DE appeared in adults between 145–165 ms. Post-hoc Tukey tests revealed that the DE was stronger in the Numerical (Tukey p = 0.0002) than in the Physical task (p = 0.0241). Between 190–230 ms DEs were detected at electrode P8 in all groups, between 216–234, 210–230, and 190–210 ms in G3, G5, and adults, subsequently. In adults there was a Distance × Task interaction. The DE was marginally significant in the numerical task (p = 0.0636), and it was stronger in the physical task (p = 0.0010). Between 250–280 ms the DE was highly significant in both tasks (Numerical: p = 0.0002. Physical: p = 0.0007).

**Table 4:** Congruency effects in EEG subjects in the reaction time (A) and in the peak latency of the P3b (B). Facilitation (neutral vs. congruent) and interference (neutral vs. incongruent) effects were tested by post-hoc Tukey contrasts from the Congruency ANOVAs. Distance effects in the amplitude of ERPs at electrode P8. Results for time intervals demonstrating significant effects are shown. Distance 5 minus distance 1 (D5-D1) values are given for both tasks, in μV. The F and the p values for the distance effect are given in the two lowest rows. The results of relevant post-hoc tests are given in the text. (Distance × Task df = 1,9.)

**Groups**	**Grade 3**		**Grade 5**		**Adults**		
**Interval (ms)**	216-234	270-300	210-230	270-290	145-165	190-210	250-280

**Numerical: D5-D1**	-2.12	2.28	-1.16	1.69	1.3	-0.54	1.79
**Physical: D5-D1**	-0.43	3.89	-1.11	1.06	0.37	-1.13	1.05
**Distance: F(1,9) =**	5.93	7.34	6.21	5.31	6.33	27.53	19.49
**Distance: p**	0.0376	0.0240	0.0342	0.0466	0.0330	0.0005	0.0017
**Distance × Task: F**	--	--	--	--	40.38	4.98	10.15
**Distance × Task: p**	0.3	0.3	1.0	0.5	0.0001	0.0525	0.0111

#### Distance effect: ERP effects at all electrodes

**Figure 5:**
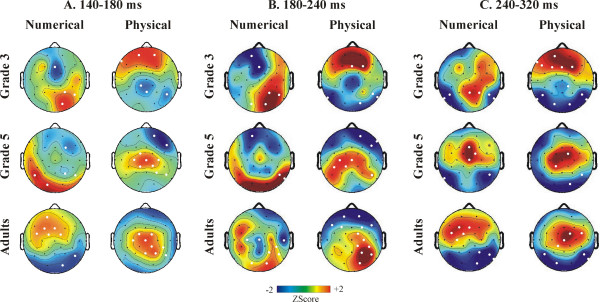
The topography of the event-related potential distance effect in normalized amplitude maps. The topography of Distance 5 minus Distance 1 difference potentials is shown in the numerical and physical tasks. White dots denote electrodes with significant distance effects (p < 0.025) between 140–180, 180–240 and 240–320 ms.

The full topography of ERP DEs (Distance 5 minus Distance 1 difference potentials) between 140–180, 180–240, and 240–320 ms is shown in Fig. 5. Group effects and Group × Electrode interactions in the difference potentials were insignificant (p > 0.3) in all time intervals in the numerical task. In the physical task the Group effect was not significant (p > 0.8). In contrast, the Group × Electrode interaction was significant between 180–240 (F(22,297) = 2.15; ε = 0.365; p = 0.0362) and 240–320 ms (F(22, 297) = 2.49; ε = 0.338; p = 0.0189), and marginally significant between 140–180 ms (F(22, 297) = 2.08; ε = 0.304; p = 0.0554). Therefore Group × Location (frontal, central, parietal) × Hemisphere (left vs. right) × Extremity (lateral vs. midline electrodes) ANOVAs were run for all three time intervals. There were Location × Group interactions between 140–180 ms (F(4,54) = 5.57; ε = 0.688; p = 0.0036), 180–240 ms (F(4,54) = 5.57; ε = 0.688; p = 0.0036), and 240–320 ms (F(4,54) = 4.16; ε = 0.654; p = 0.0158). Between 240–320 ms there was an additional Group × Location × Extremity interaction (F(4,54) = 4.38; ε = 0.855; p = 0.0063). Between 140–180 ms and 180–240 ms the topography of the DE differed in G3 relative to G5 and adults at frontal and parietal electrodes: amplitudes was more positive frontally and more negative parietally in G3 than in other groups. Between 240–320 ms both child groups had a different topography of the DE than adults (see Fig. 5.).

#### Distance effect: ERSP results

**Figure 6:**
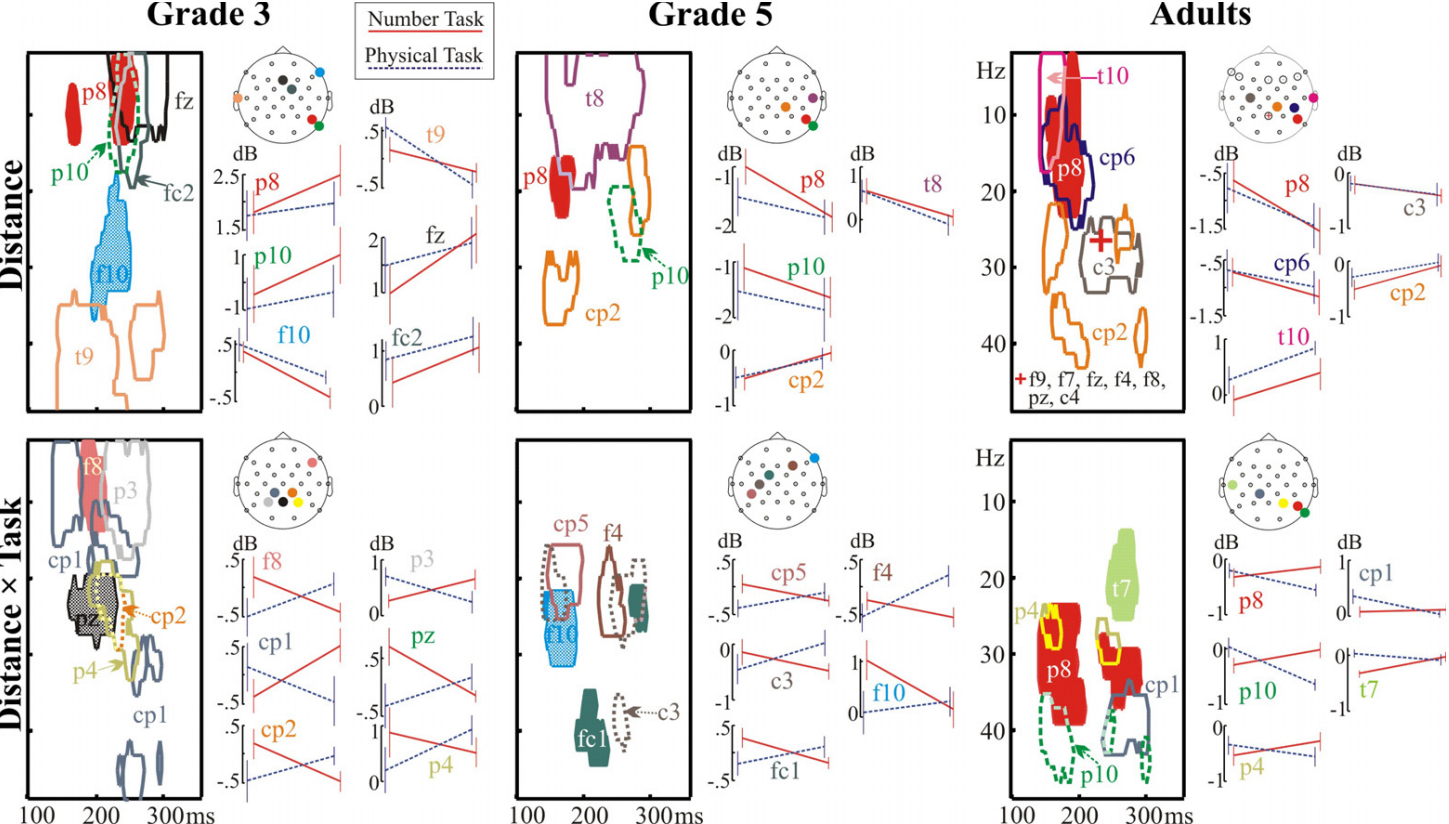
The distance effect (above) and the distance × task interaction (below) in event-related spectral perturbations (ERSP). Contour lines border significant effects (p < 0.005) in the time-frequency landscape. Graphs show the ERSP averaged for all points demonstrating significant effects at each relevant electrode. The cross in the panel for the distance effect in adults depicts a small area effect present at many electrodes.

ERSP DEs are shown in Fig. 6. (p < 0.005). In both the adults and in G5 the DE focused on a right temporo-parietal electrode cluster. G3 had a wider distribution of effects, but they too, showed DEs at the right parietal electrodes P8 and P10. Similarly to ERPs, all groups showed a DE at electrode P8. The topography of the Distance × Task interaction was more variable across groups than that of the DE. Remarkably, while both a DE and a Distance × Task interaction was detected in adults at electrode P8, the main effect and the interaction engaged very different points of the time-frequency landscape.

#### Congruency effects: the peak latency of the P3b ERP component

**Figure 7:**
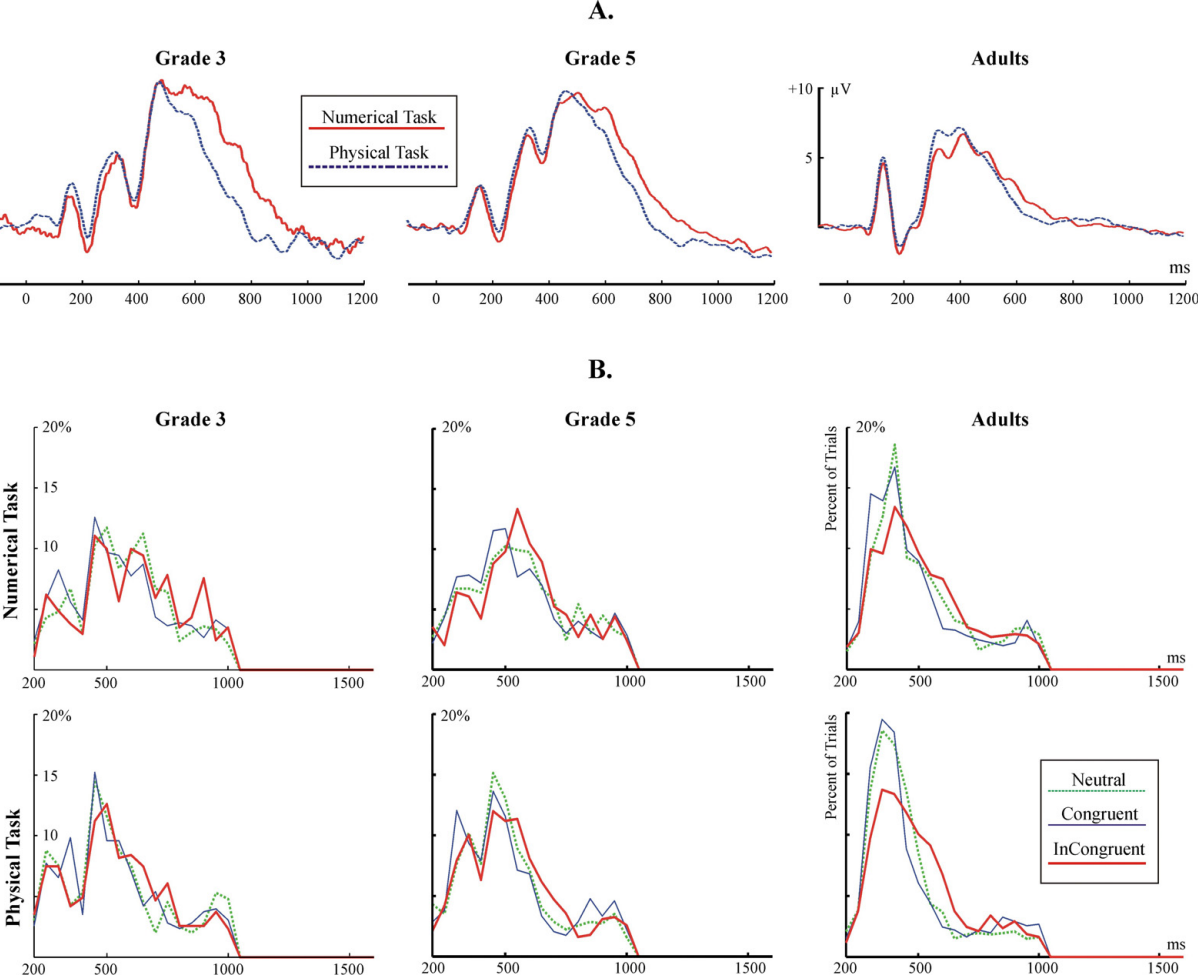
**(A) **The P3b ERP component at electrode Pz. **(B) **The distribution of the peak latency of the P3b ERP component in single trials. The percent of trials in each 50 ms bin is shown.

The grand average P3b is shown in Fig. 7A. The distribution of the peak latency of the P3b in individual trials is shown in Fig. 7B. Facilitation and interference effects in the amplitude of the P3b are visualized in Fig. 3B. Related statistics is summarized in Table 3B. There were no Congruency effects in the latency of the P3b in children. In contrast, Congruency was a significant factor in adults: there was significant facilitation and interference in the Numerical task, and interference in the Physical task.

Group effects were examined by Group × Measure (RT vs. P3b latency) ANOVAs. In the numerical task, facilitation effects in the RT and in the P3b latency did not differ by Group. However, interference effects in the RT and in the P3b latency differed significantly by group (F(2,27) = 47.84; p < 0.0001.). Post-hoc Group × Measure contrasts revealed that the RT vs. P3b difference was significant in children but not in adults (G3: p = 0.0006. G5: p = 0.0006. Adults: p = 0.3.). In the physical task, interference in the RT and in the P3b latency differed by group (F(2,27) = 35.36; p < 0.0001). Post-hoc Group × Measure contrasts revealed that the RT vs. P3b difference was significant in G3 (p = 0.0002), marginal in G5 (p = 0.0668), and insignificant in adults (p = 0.6.).

In children, the P3b latency was shorter than the RT in all cases (Tukey p: 0.0001<p < 0.03). In adults, the P3b latency was significantly shorter than RT in the Neutral (p = 0.0423) and Incongruent (p = 0.0156) conditions of the numerical task, but not in other cases.

#### Task effects: the amplitude of the P3b ERP component

The grand average P3b onset at about the same time in both tasks, but its offset seemed to be longer in the numerical than in the physical task. Therefore the mean amplitude of the P3b was examined in each 100 ms long interval between 500–900 ms by Task × Congruency ANOVAs. In adults the amplitude of the P3b was more positive between 600–700 ms in the numerical than in the physical task (1.95 ± 1.06 μV vs. 0.86 ± 1.42 μV; F(1,9) = 6.13; p = 0.0351). In G5 the amplitude of the P3b was more positive in the numerical than in the physical task between 600–700 ms (F(1,9) = 4.61; p = 0.0603), 700–800 ms (3.23 ± 2.26 vs. 1.16 ± 2.43 μV; F(1,9) = 5.63; p = 0.0412), and 800–900 ms (0.83 ± 1.45 vs. -0.92 ± 2.06 μV; F(1,9) = 7.01; p = 0.0265). In G3 the amplitude of the P3b was more positive in the numerical than in the physical task between 600–700 ms (8.52 ± 2.27 vs. 4.75 ± 2.37 μV; F(1,9) = 7.23; p = 0.0248), 700–800 ms (5.26 ± 2.63 vs. 1.27 ± 2.18 μV; F(1,9) = 10.24; p = 0.0108), and marginally between 800–900 ms (F(1,9) = 4.42; p = 0.0647).

#### Congruency effects: the lateralized readiness potential

**Figure 8:**
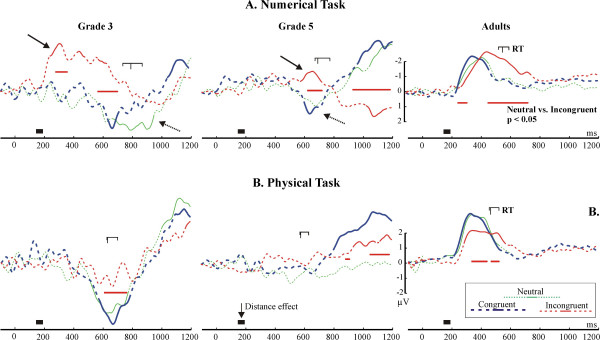
The Lateralized Readiness Potential (LRP). LRP curves are represented by solid lines where the LRP significantly deviated from zero. The dotted arrows show the opposite polarity of the LRP in children relative to adults in the neutral and congruent conditions. The solid arrow shows the incorrect response tendency signalled the LRP in the incongruent condition of numerical comparison. The red horizontal markers denote significant differences between LRPs in the neutral vs. incongruent conditions. The timing of the first EEG distance effects is represented by thick horizontal markers at the time axes. The reaction time is marked above the LRP curves ("RT"). The long vertical marker denotes the reaction time in the neutral condition; the shorter markers denote the reaction time in the congruent (earlier) and incongruent (later) conditions.

The LRP is depicted in Fig. 8. Adults showed the expected pattern of the LRP: the behavioural response was preceded and accompanied by negative polarity LRPs. In the numerical condition the LRP significantly deviated from the baseline at around 225 ms in the neutral and congruent conditions, and at 275 ms in the incongruent condition (began to rise at 250 ms). In the physical task the LRP significantly deviated from the baseline at 220 ms in the congruent and neutral conditions, and at 280 ms in the incongruent condition (began to rise at 230 ms). Children demonstrated an opposite pattern relative to adults: the behavioural response was accompanied by a positive-going LRP (dotted arrows in Fig. 8. In G3, the deviation of the LRP into the positive direction started only after 450 ms, and the deviation reached significance only after 500 ms. In G5, visible deviations started and reached significance only after 500 ms. The LRP could not be reliably identified in G5 in the Physical task. There were two conspicuous differences between adults and children. First, the LRP on-set and peaked much later in children than in adults. Second, in adults there was no sign of a dominant incorrect response in the incongruent condition: the LRP went into the negative direction in all conditions without ever turning positive. In contrast, in G3 the LRP in the incongruent condition of the Numerical task began to go into a negative direction at around 190 ms. It became more and more negative until 300 ms, than it gradually returned to the baseline until 800 ms (RT = 870 ms), and became positive. There was a similar but less expressed effect in G5, starting 200 ms before the RT, and peaking 100 ms before the RT. This negative deflection was present in neither the congruent, nor in the incongruent condition.

## Discussion

### The distance effect in ERPs and in the ERSP

We have successfully replicated and extended the findings of Temple and Posner in the NSP [12]. On the average, Grade 5 children responded 140 ms slower than adults, and Grade 3 children responded 210 ms slower than adults in the controlled and automatic numerical comparison tasks. In sharp contrast with this, both children and adults demonstrated significant EEG DEs between 140–320 ms after stimulus presentation. This was the case both when numerical information was task-relevant and when it was task-irrelevant. The timing of the ERP DE confirms the results of Temple and Posner [12], and is in line with studies demonstrating the earliest parietal DEs in adults between 124–300 ms [6,11-15]. The timing of ERSP DEs (150–300 ms) is also in agreement with an earlier study [14]. The early emergence of EEG DEs in all age groups in both the numerical and physical comparison tasks suggests that a refined representation of number was accessed with a similar speed in both adults and children, both when numerical information was task-relevant and when it was task-irrelevant [18].

The EEG DEs appeared over different electrodes in different tasks and age groups. In our study the only parietal electrode consistently demonstrating both ERP and ERSP DEs in all age groups was the right parietal electrode P8. Temple and Posner [12] found DEs over left and right parietal electrodes having approximately equivalent positions to electrodes P3, P4, P7 and P8. They noted no hemispheric interaction in the DE. Dehaene [11] reported that the DE was initially of the same size over both left and right parietal electrodes (approximating P3 and P4) between 174–198 ms, whereas the DE became larger over right than left parietal electrodes between 206–232 ms. Pinel et al. [6] detected the first trace of the DE over right temporal electrodes. Szûcs and Csépe [13,14] used acoustic stimuli, and found that the DE was more expressed over right than left parietal electrode sites. Soltész et al. [15] found DEs over both the left and right hemisphere. The ERSP DEs were present mainly over right temporo-parietal electrodes in adults and in grade 5 children, while the distribution of the effect was more variable in grade 3. The current results are in line with our former study where ERSP DEs were found with a right hemispheric predominance in both congenitally blind people and sighted adults [14]. Thus, considering all available data, the early parietal EEG DE seems to be somewhat more expressed over right than left parietal electrodes. It is an open question whether these hemispheric effects reflect the differential contribution of the left and right parietal cortex to magnitude processing.

Recent fMRI studies examining the DE in the NSP in adults [7-9] found DEs in several brain areas (left and right IPS, right superior temporal sulcus, middle temporal gyrus, superior parietal lobule, left and right precuneus, left middle temporal gyrus and in the posterior cingulate). This indicates that an extended network of brain areas take part even in simple numerical comparison. EEG effects may reflect the electric activity of any of the brain sources involved. In coherence, we found that the DE was not restricted to parietal electrodes. Furthermore, the DE reached significance over different configurations of electrodes in children and adults. However, the overall voltage distribution coinciding with the DEs was similar in all groups in numerical comparison. In contrast, in the physical comparison task there were topographic differences between children and adults between 140–180, 180–240 and 240–320 ms. This suggests that the numerical processing network was more similar between children and adults in the numerical comparison task than in the physical comparison task. A possible explanation for this is that cognitive processing was more variable when magnitude processing was covert. The topographic differences between adults and children may be explained in different ways. It is possible that the configuration of ERP generators changed from children to adults, or that the relative strength of the sources changed from childhood to adulthood, or that the anatomical positions of ERP sources changed from children to adults [36]. Recent fMRI studies of the NSP reported that children had more expressed DEs in the frontal than in the parietal cortex [25,26]. A further study also reported larger prefrontal cortex and anterior cingulate activity during mental calculation in children than in adults [37]. These findings suggest that the neural networks underlying magnitude processing undergo developmental changes. The following results also suggest that frontal control processes play a larger role in the NSP in children than in adults.

#### Facilitation and interference effects in the RT/P3b and in the Lateralized readiness potential

One of our main objectives was to study the role of developing executive functions in the NSP. The amount of interference in the RT and in the peak latency of the P3b did not differ in adults. However, in children, the interference in the RT was much larger than the interference in the latency of the P3b. This suggests that the interference onset later in children than in adults. Most probably, interference was more related to response than to stimulus-processing in children as compared with adults [28-30]. The lengthy response-processing in children is also reflected in the fact that the RT-P3b latency difference was much larger in children than in adults. The above observations confirm the hypothesis of Temple and Posner [12], according to which children perform slower in numerical comparison than adults because of their less developed ability to organize behavioural responses. The improvement of the ability to respond effectively is probably related to the development of executive functioning and behavioural inhibition, usually associated with the prolonged maturation of the frontal lobes [24,38,39].

The LRP data not only confirms the P3b/RT data but also sheds light on the nature of response-related difficulties in children. In adults the LRP appeared as expected: A negative-going LRP started at around 220 ms and coincided with the RT [32]. In contrast, a significantly positive-going LRP coincided with the RT in children. To our knowledge no LRP data has been published on children. Therefore, considering the relationship of the LRP and the RT, we assume that the correct motor response was preceded and accompanied by a positive-going LRP in children. It would be premature to interpret the polarity reversal of the LRP in children. Nevertheless, its relationship to the RT seems to be clear. The LRP accompanied the RT on-set and deviated significantly from baseline more than 250–300 ms later in children than in adults. This is in sharp contrast with the EEG DE which emerged at around 200 ms in both adults and children. The LRP attests that contrary to the early availability of numerical information children needed more time than adults to organize a successful motor response. This confirms the P3b/RT data.

Besides confirming that the organization of responses was generally slower in children, the LRP provides additional evidence that in the numerical task a major problem of children was the inadequate inhibition of incorrect response tendencies. In adults there was no sign of a dominant incorrect response tendency in the incongruent condition. In contrast, there was an opposite to normal deflection of the LRP in the incongruent condition of the numerical task in grade 3, and less conspicuously in grade 5 children. This seems to be a clear sign of an initial incorrect response tendency in children. This incorrect tendency appeared when the irrelevant physical magnitude information conflicted with the task-relevant numerical magnitude information. The appearance of the incorrect tendency was probably the consequence of the faster processing of physical than numerical information (the P3b offset later in the numerical than in the physical comparison, and the RT was shorter in the physical than in the numerical comparison): The faster processed physical information initially caused a tendency to respond with the incorrect hand in children.

Convergent brain imaging data confirms that children between the ages of 8–12 years have immature prefrontal inhibition function relative to adults [40-44]. In our study children were as accurate as adults, and the LRP also returned to its normal polarity. Therefore it is evident that children could rectify the incorrect response tendency and finally pressed the correct response button. This is in-line with data demonstrating that children more than 6 years old can successfully exploit sufficiently prolonged response periods for maintaining their accuracy under demanding task requirements [23,45,46]. Our data most probably captures the LRP manifestation of these self-correction mechanisms. The less pronounced nature of the incorrect tendency in grade 5 than in grade 3 children is most probably the consequence of a developmental trajectory of response-organization skills. Speculatively, our results suggest that immaturities of behavioural inhibition abilities, or frontal control processes in general may significantly contribute to arithmetic performance under time pressure [47,48], and to the appearance of certain developmental arithmetic disabilities in children. For example, we have recently found that adolescents with developmental dyscalculia were impaired in executive functioning, probably guiding attention to numbers and directing the focused processing of arithmetic information [15]. Further, a recent study found an atypical facilitation/interference pattern in the NSP in children suffering from attention deficit hyperactivity disorder [49], which is often associated with arithmetic deficits.

#### The behavioural reversed distance effect in the physical comparison task

All groups showed a normal behavioural DE in numerical comparison, and a similar pattern of a reversed DE with a similar effect size (in milliseconds) was found in physical comparison in all groups. The similar pattern of the reverse DE in adults and children was confirmed by the lack of a group × distance interaction in physical comparison. However, when the age groups were tested separately, the reversed DE did not reach significance in children. Some papers have reported a reversed or normal DE in physical comparison, while others reported no DE. Henik and Tzelgov ([17] Exp. 2.) found a reversed DE in the incongruent condition of physical comparison in adults. Girelli et al. [21] reported a reversed DE both in adults and in grade 5 children, but not in grade 1 and 3 children (using numerical distances 1 and 5). Kaufmann et al. found a normal DE in the physical comparison task in both adults [8] and children [25] (using numerical distances 1 and 4). In contrast with the above results, Rubinstein et al. [20,22] did not detect a DE (using numerical distances 1, 2 and 5 in Ref 20; and distances 1, 2 and 4 in Ref. 22). Our data is in line with the reversed DE found by Girelli et al. [21] and by Henik and Tzelgov [17]. The pattern of the reversed DE can be explained by considering the interactions between the properties of the neural representation of number, task requirements, physical and numerical comparison processes, and comparison and response-related processing.

In the numerical comparison task subjects discriminated the digits on the basis of their numerical meaning, and a normal DE appeared: The larger the difference between the digits, the easier it was to discriminate between them. The normal DE is explained by the concept of an analogue-like magnitude representation [4], best described by the neuronal model of Dehaene and Changeaux [50]. The model hypothesizes that number meaning is represented by differentially tuned neuronal populations giving maximal response to their preferred numerosity. Neuronal populations do not only respond to their preferred number, but also to adjacent numerosities. The normal DE is a consequence of the larger overlap between the neural representations of closer than further away numbers. The model has been supported by single-cell studies in monkeys [51,52] and by a recent imaging study [10]. The above model can easily explain the reversed DE. In the physical comparison task numerical meaning was irrelevant. Nevertheless, the ERPs and ERSP DEs attest that task-irrelevant numerical information was processed both in children and in adults. It is reasonable to assume that if the neural representation of the (task-irrelevant) numerical magnitude of the two digits overlapped to a large extent (small numerical distance), they would facilitate each other's perception more relative to the case when this overlap was smaller (large distance). Hence, the closer the numerical magnitude of the numbers, the faster was their involuntary processing, and the further away were the magnitudes the digits represented, the slower was their processing.

The above model can explain why a reversed DE appears when numerical information is task-irrelevant, provided that the physical size difference between the digits is the same in the case of both large and small numerical distance. However, if the physical size distance between the digits is not constant then numerical and physical discrimination processes [53] and the resulting facilitation/interference effects will interact with each other. For example, Kaufmann at et al. [8,25] found a normal behavioural DE in physical size comparison. However, they used two different physical size distances in case of small and large numerical distance: Small numerical distance was always coupled with large physical size distance, and large numerical distance was always coupled with small physical size distance. This means that the physical size information contradicted the numerical information more in the case of small than larger numerical distance. Hence, interference was larger when the numerical distance was small relative to the case when the numerical distance was large. The larger interference in the small distance condition could slow down processing relative to the speed of the large distance condition. This slowing down can be interpreted as a "normal distance effect". In contrast, in our experiment the physical size distance between digits was constant for both numerical distances. Therefore the interference did not co-vary with numerical distance in such an extent as in the experiments of Kaufmann et al. [8,25], and a reverse DE appeared.

#### The role of response-related processing in behavioural effects

The interaction of response-related and numerical processing should also be considered when interpreting distance and congruency effects in the NSP. First of all, similarly to some earlier findings [17,21], the reversed DE was stronger in the incongruent than in the congruent condition of the physical task in our experiment. Most probably, this can be explained by the stronger response interference in the incongruent than in the congruent condition: The strong interference slowed down responding which allowed the covertly evaluated numerical information to affect the reaction time in the incongruent condition. Further, it is reasonable to believe that the less expressed reversed DE in children than in adults was the consequence of an interaction between response-related and numerical processing: The P3b and LRP data demonstrated that response organization in children was less effective than in adults. This suggests that after the numerical information became available, it was harder for this information to activate a motor response in children than in adults, and that it was harder for the task-irrelevant numerical information to affect RT in children than in adults. Hence, it was also harder for the reverse DE to appear in the RT, except in the incongruent condition where responding was slow.

Between-group differences in response-related processing should also be considered when interpreting the facilitation/interference pattern in the NSP. The physical comparison task is particularly interesting. In this task the presence or absence of facilitation and interference effects is usually interpreted as indicating that the irrelevant numerical dimension of the stimuli has been processed. Hence, this task is being used as a measure of automatic activation of numerical information. One previous study reported a facilitation effect in physical size comparison in grade 3 children, grade 5 children and adults, but not in grade 1 children [22]. Another study did not detect a similar facilitation effect [21]. In our study we did not find a facilitation effect in children and found only a marginal facilitation effect in adults. One explanation for the lack of the facilitation effect in children would be that children process numerical information less automatically than adults. However, in our study the EEG DE appeared with the same time course in both children and adults. This suggests that children did process numerical information automatically in the physical comparison task. Therefore the most probable explanation is that numerical information did not result in behavioural facilitation because of ineffective response organization in children. The usually very small behavioural facilitation effect in the physical comparison task may be particularly sensitive to the modulating effect of response-related processes.

With regard to interference, Girelli et al. have reported that the amount of interference increased from grade 3 to 5 in both numerical and physical size comparison [21]. This was explained by assuming that the integrity of the numerical and physical size dimensions increased from grade 3 to 5. Another study did not explicitly report a similar effect [22]. In contrast to Girelli et al. we found that the amount of interference did not change from grade 3 to grade 5. Further, in line with Girelli et al. we also found that the amount of interference decreased from grade 3 and 5 to adults. Rubinstein et al.'s findings also seem to be in line with this (see Figure 1 in Ref. [22]). The decreasing pattern of interference from childhood to adulthood is in line with studies demonstrating that children are more susceptible to interference than adults [40,41,44]. One explanation for this would be that the irrelevant physical information has a larger perceptual saliency for children than for adults [54]. However, if the saliency of the physical information had been larger than that of numerical information, then the interference from the irrelevant numerical information would have been smaller in younger than in older children in the physical comparison task. This was clearly not the case. Further, the ERP DE had the same timing in children and adults. This implies that the numerical information had a similar saliency, and was automatically processed with a similar time course in both children and in adults. Our data suggest that a crucial factor behind the larger interference in children was not the differential saliency of the stimulus dimensions, but rather, the stronger response-interference in children than in adults. This strong response-interference in children resulted in the stronger behavioural interference effects observed in children compared with adults.

In summary, the lack and/or the level of significance of the reversed DE and of the behavioural facilitation effect in the physical comparison task depends on several factors, including the interaction between the comparison of exact stimulus parameters, their interaction with facilitation/interference processes, and the interaction of numerical and response-related processing. Therefore differences in the pattern of interference cannot be attributed to differences in numerical or physical magnitude processing alone. For example, a larger interference effect in children than in adults can be a consequence of children's less developed response abilities rather than that of their different magnitude processing relative to adults. This suggests that caution is needed when drawing conclusions about numerical processing skills by interpreting purely behavioural measures of the NSP.

### Summary

We found that children discriminated magnitude slower than adults in the NSP. In contrast, ERP and ERSP DEs appeared in both numerical and physical comparison in both children and adults between 140–320 ms after stimulus presentation. This attests that a refined representation of number was activated with a similar speed in all age groups both in controlled and automatic magnitude comparison. The analysis of the latency of the P3b ERP component (measured in single trials) and LRP data suggest that interference effects were more related to response than to stimulus processing in children compared with adults. The LRP data revealed that the inhibition of incorrect response-tendencies was an important source of interference in children. We conclude that despite a similar behavioural profile in children and adults, partially different cognitive processes underlie their performance in the NSP. These cognitive differences should be considered when measuring the automaticity of numerical processing in children. A reverse DE appeared in physical comparison. This was explained by current models of number processing. Behavioural effects in the NSP depend on interactions between comparison, facilitation/interference and response-related processes. This suggests that caution is needed when interpreting purely behavioural measures of the NSP.

## Competing interests

The author(s) declare that they have no competing interests.

## Authors' contributions

DSz and FS designed the experiment. FS programmed the paradigm. FS collected experimental data. EJ collected and analyzed IQ and calculation data. DSz carried out the major part of experimental data analysis. FS contributed significantly to experimental data analysis. DSz and FS designed and prepared illustrations. DSz wrote the article. VCs gave advice, provided labspace and intellectual support.
